# An Important Role for Syndecan-1 in Herpes Simplex Virus Type-1 Induced Cell-to-Cell Fusion and Virus Spread

**DOI:** 10.1371/journal.pone.0025252

**Published:** 2011-09-21

**Authors:** Ghadah A. Karasneh, Mohamed Ali, Deepak Shukla

**Affiliations:** 1 Departments of Ophthalmology and Visual Sciences, University of Illinois at Chicago, College of Medicine, Chicago, Illinois, United States of America; 2 Departments of Microbiology and Immunology, University of Illinois at Chicago, College of Medicine, Chicago, Illinois, United States of America; Mayo Clinic, United States of America

## Abstract

Herpes simplex virus type-1 (HSV-1) is a common human pathogen that relies heavily on cell-to-cell spread for establishing a lifelong latent infection. Molecular aspects of HSV-1 entry into host cells have been well studied; however, the molecular details of the spread of the virus from cell-to-cell remain poorly understood. In the past, the role of heparan sulfate proteoglycans (HSPG) during HSV-1 infection has focused solely on the role of HS chains as an attachment receptor for the virus, while the core protein has been assumed to perform a passive role of only carrying the HS chains. Likewise, very little is known about the involvement of any specific HSPGs in HSV-1 lifecycle. Here we demonstrate that a HSPG, syndecan-1, plays an important role in HSV-1 induced membrane fusion and cell-to-cell spread. Interestingly, the functions of syndecan-1 in fusion and spread are independent of the presence of HS on the core protein. Using a mutant CHO-K1 cell line that lacks all glycosaminoglycans (GAGs) on its surface (CHO-745) we demonstrate that the core protein of syndecan-1 possesses the ability to modulate membrane fusion and viral spread. Altogether, we identify a new role for syndecan-1 in HSV-1 pathogenesis and demonstrate HS-independent functions of its core protein in viral spread.

## Introduction

Herpes simplex virus type-1 (HSV-1) is a worldwide health problem that causes a wide range of diseases. It is a leading cause of infectious corneal blindness in the developed world and sporadic, fatal encephalitis worldwide. The virus also causes asymptomatic life-long infections in a majority of adult human population and uses a clever way of spreading from cell-to-cell to avoid detection by the host immune system [1], [2], [3]. Absence of an effective vaccine or microbicide against latent or recurrent HSV, and the fast emerging drug-resistant virus isolates highlight the need for developing new antivirals for HSV-1 [4]. Therefore, characterizing the molecular basis of HSV-1 entry into host cells and the viral-cellular interactions involved in viral spread are crucial for the development of new approaches to prevent the infection.

HSV-1 follows different entry routes depending on the type of the cell it infects [5], [6], [7]. It can fuse at the plasma membrane, enter via endocytosis, or get captured by cells in a phagocytosis-like manner and fuse with the phagosomal membrane [6], [7], [8]. Five HSV-1 glycoproteins are known to be involved in HSV-1 entry, and these are HSV-1 glycoproteins gB, gC, gD, gH, and gL [5], [7], [8]. The glycoprotein gC is not essential for entry, and in its absence the virus can still enter the host cell [9]. Interaction between the viral envelope and the plasma membrane starts with the attachment of the virus through its glycoproteins gB and gC to heparan sulfate (HS) moieties of HS proteoglycans (HSPG) on the surface of a host cell [10]–[13]. Next, a third glycoprotein, gD binds to one of its receptors, nectin-1, herpesvirus entry mediator (HVEM), or 3-O sulfated HS [14], [15], [16] to start the process of membrane fusion and penetration. Binding of a cell surface receptor to gD is a necessary step for entry of HSV-1. Fusion of the viral envelope with the host cell membrane then follows with the combined action of HSV-1 gD, gD receptor, gB, gH, gL [14], and possibly gB receptors [17], [18] and gH receptors [19].

A similar process of membrane fusion termed HSV-1 induced cell-to-cell fusion, involving the fusion of plasma membrane of an infected cell with that of a neighboring uninfected cell, is thought to occur during cell-to-cell spread [8]. Upon virus entry, viral glycoproteins are expressed on the surface of infected cells. This allows the binding and fusion of the viral glycoproteins on the surface of infected cells with neighboring uninfected cells, forming syncytia [14]. Cell-to-cell fusion allows the virus spread into surrounding cells without the need to be released outside the cell, allowing efficient transmission and escaping the host immune system. The spread of HSV-1 is relatively poorly understood and virtually nothing is known about the role of HSPGs in this process.

Syndecans are single transmembranous heparan sulfate proteoglycans (HSPG) with the HS chains covalently attached to the extracellular portion of the core protein [20]. Syndecans family constitutes the most abundant HSPGs expressed on the surface of mammalian cells [21], [22], [23]. Four members in the syndecan family have been described in the mammalian cells (syndecan-1 to 4). The syndecan core protein is linearly organized into three regions: the N-terminal ectodomain that is unique for each syndecan, conserved transmembrane domain, and the cytoplasmic domain that consists of two conserved regions and one variable region specific for each syndecan [20], [23]. The ectodomain has HS attachment sites. *In vivo* studies have shown that syndecans-1-2 and -3 are expressed on specific cell types. For example, syndecan-1 is expressed predominantly in epithelial and mesenchymal tissues, syndecan -2 in cells of mesenchymal origin, neuronal and epithelial cells, and syndecan-3 in neuronal and musculoskeletal tissue, whereas syndecan-4 is expressed in virtually every cell type [24], [25].

Previous studies on the role of HSPG have been primarily focused on the role of HS chains as an attachment receptor for HSV-1, while the core protein was given a passive role of carrying the HS moieties. However, recent work from our lab and others [10], [11] has shown that the syndecan family of HSPG is directly involved in HSV-1 entry. The aim of this study was to investigate the role of syndecan-1 in membrane fusion and cell-to-cell spread of infectious virus. Using wild type CHO-K1 cells and the mutant CHO-745 cells deficient in glycosaminoglycans (GAGs) synthesis [26] we show that syndecan-1 is important for HSV-1 induced membrane fusion and cell-to-cell spread of the virus in HS-independent manner. CHO-745 cells have an inactive form of the xylosyltransferase enzyme essential for GAG synthesis. Therefore, these cells express only the core protein of syndecan-1 without any of the GAGs including HS.

In addition, using plaque assays performed in methylcellulose, which restricts virus spread through the medium allowing plaque formation due to virus spread from cell-to-cell, we show syndecan-1′s role in HSV-1 cell-to-cell spread in human corneal epithelial (HCE) cells, a natural target for HSV-1 infection. Evidence has shown that syndecan-1 exhibits very strong localization within the corneal epithelium that represents one of the major infection sites for HSV-1 that may precede infection of other sites within the eye [27], [28], [29]. We also demonstrate that the downregulation of syndecan-1 results in fewer plaques and therefore, less infectious virus production. Overall, our study demonstrates a new role for syndecan-1 in HSV-1 cell-to-cell fusion and spread.

## Results

### Syndecan-1 downregulation does not affect cell viability

**Figure 1:**
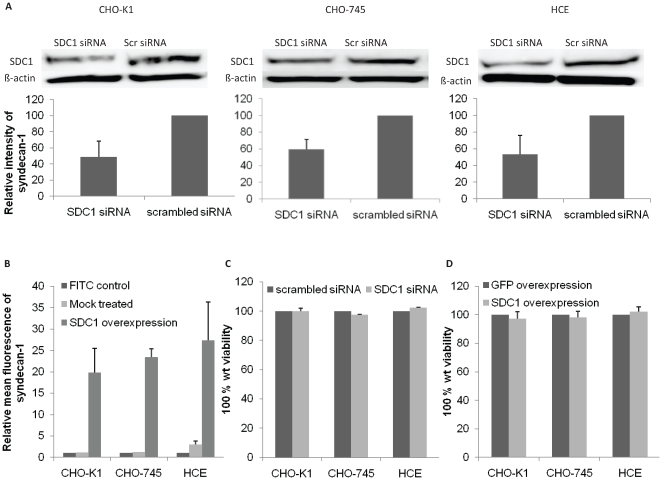
Syndecan-1 knockdown or overexpression do not affect cell viability. (A). CHO-K1, CHO-745, and HCE cells were transfected with scrambled (scr) siRNA or syndecan-1 (SDC1) siRNA. 72–96 h after transfection, immunoblots of cell lysates were prepared and probed with anti-SDC1 polyclonal Ab. β-actin protein level was measured as loading control. Representative blots are shown. Protein bands were quantified using ImageQuant TL image analysis software (version: 7). SDC1 protein expression (mean ± 1SD), normalized to that of β-actin, of at least three independent experiments was quantified by calculating the relative intensity of each syndecan-1 band relative to the control scrambled siRNA treated bands, and presented as bar graph. (B) Cells were grown in 6-well plates, mock treated or transfected with human SDC1 plasmid for 48 h. Cell surface level of SDC1 was evaluated by flowcytomety. FITC stained cells were used as background control. Results are representative of two independent experiments (C, D). Cells were grown in 96-well plates, transfected with scrambled siRNA or SDC1 siRNA for 48 h (C), or transfected with control GFP plasmid or human SDC1 plasmid for 24 h (D). Triplicate wells were evaluated for cell viability using MTS assay. Results are expressed as 100% wild type (wt) viability where they represent the percent corrected absorbance after subtracting the background absorbance, relative to scrambled siRNA transfected cells (C), or relative to GFP transfected cells (D), and are mean ± 1SD of at least 2 independent experiments.

In order to understand the significance of syndecan-1 during HSV-1 infection, three cell lines were used to examine its role in different aspects of HSV-1 infection. The cell lines used were the wild type Chinese hamster ovary (CHO-K1) cells, a GAG-deficient CHO cell line (CHO-745) [30], [31], and HCE cells which are a prime target for HSV-1 infection [29]. The cell lines were subjected to syndecan-1 overexpression or selective downregulation by siRNA. While Human syndecan-1 plasmid was used to enhance syndecan-1 production, two sets of siRNAs were used to selectively knockdown its expression. The downregulation of syndecan-1 in CHO-K1, CHO-745, and HCE cells was confirmed at the protein level using Western Blot analysis. Densitometric analysis showed that treatment with syndecan-1 specific siRNA resulted in a significant decrease (approximately 50%) in protein production, confirming the specific downregulation of syndecan-1 (Fig. 1A). Increase in syndecan-1 level was confirmed using flowcytometric analysis on CHO-K1, CHO-745, and HCE cells, and resulted in approximately 20–25 fold increase. Relative mean fluorescence of syndecan-1 on the surface of CHO-K1, CHO-745, and HCE cells is shown in Fig. 1B. To evaluate whether alterations in syndecan-1 levels affect cell viability, an MTS assay was performed 3-days post syndecan-1 downregulation, and 1-day post syndecan-1 overexpression. Neither the former nor the latter affected cell viability compared to control cells that were either transfected with scrambled siRNA or transfected with the control green fluorescent protein (GFP) plasmid (Fig. 1C–D). These results demonstrated that syndecan-1 downregulation and overexpression were successful in CHO-K1, CHO-745, and HCE cells, and neither affected cell viability.

### Increase in syndecan-1 level on target cells promotes, and its loss on target cells inhibits HSV-1 induced cell-to-cell fusion

**Figure 2:**
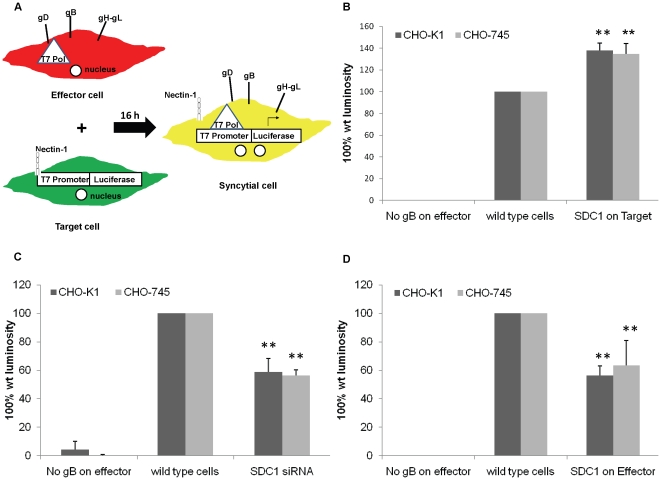
Syndecan-1 overexpression on target cells enhances cell fusion, while its overexpression on effector cells inhibits cell fusion. (A). An illustration of cell fusion assay that was exploited to understand the contribution of syndecan-1 during HSV-1 induced cell-to-cell fusion. Effector cell population that expresses HSV-1 fusion glycoproteins plus T7 polymerase is mixed with the target cell population that expresses nectin-1 as a gD receptor and the luciferase reporter gene under the control of T7 promoter. Luciferase reporter gene activity is determined to quantify cell-to-cell fusion. (B). Target cells for CHO-K1 and CHO-745 cells were either transfected with GFP control plasmid (wild type cells) or transfected with syndecan-1 plasmid and mixed with effector cells 24 h post-transfection. As a negative control, target cells were mixed with effector cells that lack HSV-1 gB (C). Target cells for CHO-K1 and CHO-745 cells were either mock treated (wild type cells) or transfected with syndecan-1 specific siRNA and mixed with effecor cells 48 h post-transfection. As a negative control, target cells were mixed with effector cells that lack HSV-1 gB. (D). Effector cells for CHO-K1 and CHO-745 cells were transfected with either GFP control plasmid (wild type cells) or human syndecan-1 plasmid and mixed with target cells 24 h post-transfection. As a negative control, target cells were mixed with effector cells that lack HSV-1 gB. (B, C, D). Fusion was measured 16 h post mixing. Results are presented as mean ± 1 SD of at least 3 independent experiments. *SDC1*, syndecan-1.

HSV-1 induced cell-to-cell fusion can be studied by co-cultivating two populations of cells: “Target” and “Effector” cell populations. Target cells express gD receptor and the luciferase reporter gene under the control of T7 promoter. Effector cells express HSV-1 glycoproteins that are absolutely required for virus fusion (gB, gD, gH, and gL) plus T7 polymerase [30]. Luciferase reporter gene activity is determined 16 h post mixing the two populations to quantify cell-to-cell fusion. As a negative control, target cells are mixed with effector cells that lack HSV-1 gB, where cell-to-cell fusion is expected to be reduced dramatically because of the absence of one of the four absolutely required HSV-1 glycoproteins for cell-to-cell fusion; gB (Fig. 2A). Using this co-cultivating system, we aimed to understand the contribution of the core protein of syndecan-1 during HSV-1 induced cell-to-cell fusion. Two cell lines were used, the wild type CHO-K1 and a GAG-deficient CHO cell line CHO-745 that has an inactive form of xylosyltransferase enzyme, which is required for the initiation of GAG chain by transferring xylose to the GAG core protein [30], [31]. We have previously shown that HS plays negative role in cell fusion; as cell-to-cell fusion increases in the absence of HS on the cell surface [26]. Thus using CHO-745 cells enables the elimination of the effect of HS chains on cell fusion. Enhancement of syndecan-1 on target CHO-K1 and CHO-745 cells resulted in a significant increase (37.9±6.9% and 34.5±9.9% increase respectively *P*<0.0001) in cell-to-cell fusion compared to wild type cells that were transfected with GFP control plasmid (Fig. 2B). Moreover, Syndecan-1 downregulation on target CHO-K1 and CHO-745 cells inhibited HSV-1 induced cell-to-cell fusion (41.3±9.5% and 43.8±4.3% inhibition respectively *P*<0.0001) compared to wild type cells that were transfected with control scrambled siRNA (Fig. 2C). These results suggest that syndecan-1 may play a role during HSV-1 induced cell-to-cell fusion, and this role is independent of HS chains.

### Enhanced syndecan-1 on effector cells inhibits cell-to-cell fusion

The fusion of the virus envelope with the host cell plasma membrane results in the expression of the virus glycoproteins on the surface of the infected cell along with the cell membrane proteins including syndecan-1. To understand the effect of increase in syndecan-1 level with the virus glycoproteins on the same cell, we overexpressed syndecan-1 on the effector cell population that also expresses HSV-1 fusion glycoproteins. Interestingly, syndecan-1 overexpression on effector CHO-K1 and CHO-745 cells inhibited HSV-1 induced cell-to-cell fusion significantly compared to wild type cells that were transfected with GFP control plasmid (*P*<0.0001) (Fig. 2D). This result was observed in CHO-K1 cells (43.6±6.0% inhibition) as well as CHO-745 cells (36.4±17.5% inhibition) that lack all GAGs including HS, suggesting that the observed inhibition is HS independent.

### Enhancement of syndecan-1 production in target cells induces the syncytial cell formation, whereas the same on effector cells inhibits syncytial cell formation and reduces the average number of nuclei per syncytia

**Figure 3:**
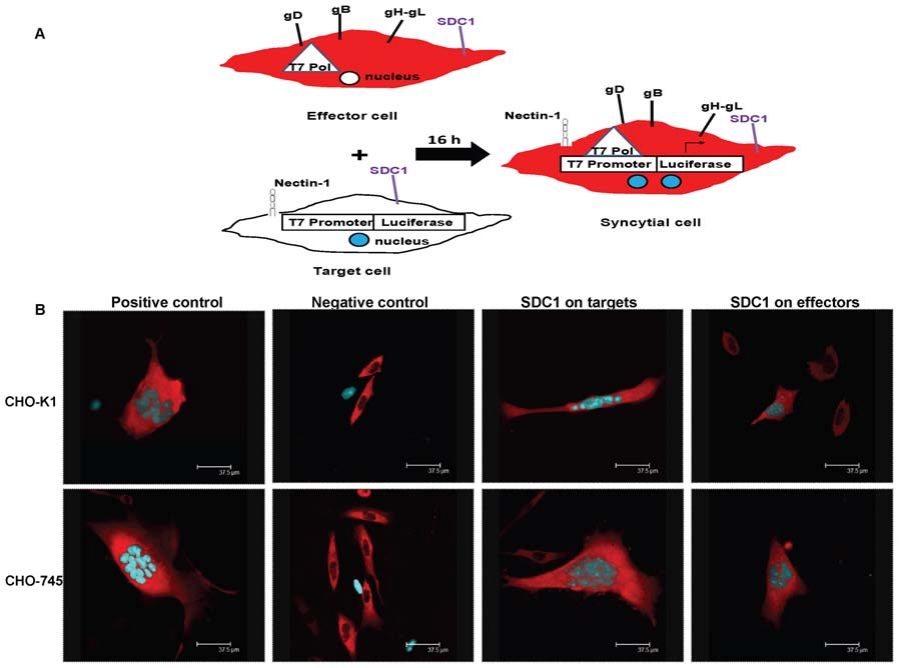
Syncytial cell formation in CHO-K1 and CHO-745 after syndecan-1 overexpression. (A). An illustration of syncytia assay that was exploited to understand the contribution of syndecan-1 during HSV-1 induced cell-to-cell fusion. Effector cell population that expresses HSV-1 fusion glycoproteins, T7 polymerase as well as a RFP-NES construct that restricts the expression of RFP to the cytoplasm is mixed with the target cell population that expresses nectin-1 as a gD receptor, the luciferase reporter gene under the control of T7 promoter as well as a CFP-NLS construct that restricts the level of CFP to the nucleus. (B). Syncytia formation was observed in cells 72 h after the mix of target cells with effector cells. *Top panels* show representative syncytia formed in CHO-K1 cells after overexpressing syndecan-1 in target, or effector cell. *Bottom panels* show representative syncytia formed in CHO-745 cells after overexpressing syndecan-1 in target, or effector cells. Positive controls are target cells mixed with effector cells where both populations have the wild-type level of syndecan-1 on the surface. Negative controls are target cells mixed with effector cells missing gB. (Scale bar = 37.5 µm). *SDC1*, syndecan-1.

HSV-1 induced cell-to-cell fusion results in the formation of large, multinucleated syncytial cells [8]. To compare the number and size of syncytia after overexpressing syndecan-1 on target or effector cells, a cyan fluorescent protein (CFP) construct attached to a nuclear localization signal (NLS) for limiting the CFP to the nuclei was additionally transfected into target cells. Likewise, the effector cells were also additionally transfected with a red fluorescent protein (RFP) attached to a nuclear export signal (NES), limiting the expression of RFP to the cytoplasm [26], [32]. Syncytia were then identified as cells expressing red cytoplasm and at least one blue nucleus (Fig. 3A). The *top panels* show representative syncytia formed in CHO-K1 cells after overexpressing syndecan-1 on target or effector cells. The *bottom panels* show representative syncytia formed in CHO-745 cells after overexpressing syndecan-1 on target or effector cells. The positive controls consist of target cells mixed with effector cells where both populations express normal levels of syndecan-1. The negative controls consist of target cells mixed with effector cells missing gB, and thus no syncytia formation (Fig. 3B).

Table 1 lists the average number of syncytia formed in CHO-K1 and CHO-745 cells in each condition and the size of syncytia formed indicated by the average number of syncytia that had 2 nuclei, 3–5 nuclei, or more than 5 nuclei. Table 1 shows that overexpressing syndecan-1 on target CHO-K1 or CHO-745 cells formed a significantly greater number of syncytia than the positive control that has target and effector cells expressing normal levels of syndecan-1(*P*<0.05). However, overexpressing syndecan-1 on effector CHO-K1 or CHO-745 cells formed a significantly smaller number of syncytia compared to the positive control (*P<*0.05). The number of nuclei per syncytia cell was also examined. Overexpressing syndecan-1 on target cells did not result in a significant increase in the number of nuclei per syncytial cell. However, when syndecan-1 was overexpressed on effector cells, syncytia formed had significantly fewer nuclei per syncytial cell (*P*<0.05).

**Table 1:** Comparison of syncytia number and nuclei count after syndecan-1 overexpression in each CHO cell type.

Cell type	Condition	Average number syncytia per well	2 nuclei per syncytia	3–5 nuclei per syncytia	>5 nuclei per syncytia
CHO-K1	Positive control	142.5±9.8	30±13.9	63±14.6	50.88±16.5
	SDC1 on targets	196.8±15.0	66±7	72.7±22.0	49.6±16.7
	SDC1 on effectors	72.8±19.3	43.3±24.9	22.2±6.5	4.5±5.7
CHO-745	Positive control	213.8±4.6	38.5±17.0	89.5±3.5	85.8±25.1
	SDC1 on targets	338.3±13.8	129.3±6.7	131.3±8.8	77.8±15.9
	SDC1 on effectors	46.8±8.8	17.3±1.8	23.5±4.9	6±5.7

Average number of syncytial cells, as well as, the average number of nuclei per syncytia was counted in CHO-K1 and CHO-745 cells after overexpressing syndecan-1 on target cells or effector cells. Positive controls are target and effector cells expressing normal levels of syndecan-1. Syncytial cells were counted 72 h post mixing. Syncytia were classified as any red cell having two or more nuclei. Number of syncytia was normalized to the number of syncytia detected in the negative control wells where the effector cell population lacks gB. The average is based on results from two independent experiment performed in duplicate (mean ± 1SD).

### The ectodomain and the cytoplasmic domains of syndecan-1 are important for inhibiting HSV-1 induced cell-to-cell fusion when syndecan-1 co-exists with HSV-1 glycoproteins on effector cells

**Figure 4:**
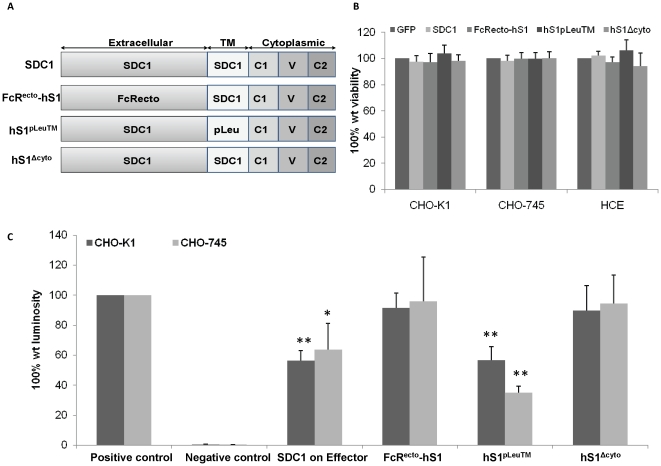
syndecan-1 ectodomain and cytoplasmic domains are important for inhibiting cell fusion when overexpressed on effector cells. (A). Syndecan-1 truncation and mutants used in the study are illustrated including the full-length wild type (*wt*) core protein syndecan-1 (SDC1) that includes an extracellular domain, transmembrane domain (TM), and COOH-terminal cytoplasmic domain. Also illustrated are the construct FcR^ecto^hS1 that is a chimera comprised of the ectodomain of human IgG Fcγ receptor Ia/CD64 fused to the transmembrane and cytoplasmic domains of human syndecan-1, the construct hS1^pLeu^™ that has the transmembrane domain replaced with leucine residues, and a truncation mutant hS1^Δcyto^ that lacks the 33 C-terminal amino acids. (B) Cells were grown in 96-well plates, transfected with control GFP plasmid, full-length *wt* human SDC1 plasmid, the construct FcR^ecto^hS1, the construct hS1^pLeu^™, or the construct hS1^Δcyto^ for 24 h. Triplicate wells were evaluated for cell viability using MTS assay. Results are expressed as 100% wild type (wt) viability where they represent the percent corrected absorbance after subtracting the background absorbance, relative to control GFP plasmid transfected cells, and are mean ± 1SD of at least 3 independent experiments. (C). Effector cells for CHO-K1 and CHO-745 cells were transfected with either control GFP plasmid, full-length *wt* syndecan-1, the construct FcR^ecto^hS1, the construct hS1^pLeu^™, or the construct hS1^Δcyto^ and mixed with the target cells 24 h post-transfection. Fusion was measured 16 h post mixing. Results are presented as mean ± 1 SD of at least 3 independent experiments. As a negative control, target cells were mixed with effector cells lacking HSV-1 gB.

To determine whether specific syndecan-1 domain(s) are required for the observed inhibition of HSV-1 induced cell-to-cell fusion when syndecan-1 is overexpressed on the effector cells along with HSV-1 fusion glycoproteins, a series of syndecan-1 molecules with specific truncations and mutations were expressed and analyzed. A diagram summarizing the mutant constructs used is shown in Fig. 4A. Cell fusion assay after overexpressing syndecan-1 mutants reveals that while the constructs FcR^ecto^-hS1 (a construct in which the ectodomain of syndecan-1 is replaced by that of the human Fc) and hS1^Δcyto^ (a construct that lacking the 33 C-terminal amino acids) were able to allow the fusion to near positive control levels that express normal levels of syndecan-1, overexpressing the construct hS1^pLeu^™ (a construct in which the transmembrane domain is replaced with leucine residues) on effector cells resulted in similar inhibition of HSV-1 induced cell fusion caused by overexpressing the wild type syndecan-1 (Fig. 4C). The cell surface expression of syndecan-1 mutants has been confirmed previously [33]. These results were observed in both CHO-K1 and CHO-745 cell lines, and were not due to cytotoxic effect of overexpressing syndecan-1 mutants, as that did not affect cell viability measured by MTS assay (Fig. 4B). These results demonstrate that syndecan-1 ectodomain and cytoplasmic domains but not the transmembrane domain are required for reduced cell fusion when syndecan-1 is overexpressed along with HSV-1 fusion glycoproteins on effector cells.

### Syndecan-1 knockdown reduces plaque formation in HCE cells

**Figure 5:**
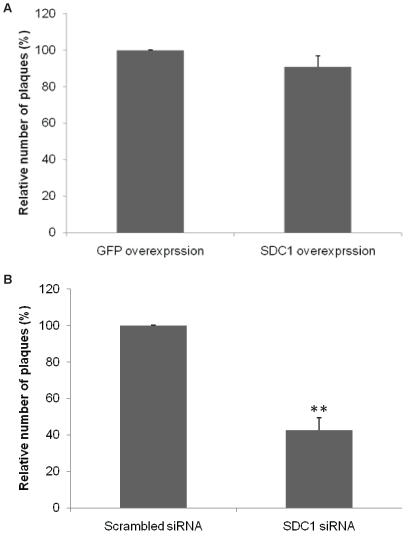
Syndecan-1 knockdown reduces plaque formation in HCE cells. (A). Monolayers of HCE cells were transfected with either control plasmid GFP, or with human syndecan-1. 24 h post-transfection cells were infected with serial dilution of HSV-1(KOS) stocks. (B). 50% confluent HCE cells were transfected with either control scrambled siRNA or syndecan-1 specific siRNA. 72 h post-transfection, cells were infected with serial dilution of HSV-1(KOS) stocks. (A, B). 72 h post-infection cells were fixed and stained with crystal violet stain. Infectivity was measured by the number of plaque forming units (PFUs). Number of PFUs was counted at the 10× objective (Zeiss Axiovert 200). Plaques consist of 15 or more nuclei were counted. Results are means ± 1 SD of three independent experiments conducted in duplicate. *SDC1*, syndecan-1.

HSV-1 has the ability to produce visible plaques on HSV-1 susceptible cells which results in central clearing as the virus spreads [34]. One way of its spread is lateral virus cell-to-cell spread by the fusion of infected cells with neighboring uninfected cells to form large multinucleated syncytial cells that can then slough off forming plaques. HCE cells were chosen to examine the contribution of syndecan-1 during HSV-1 plaque formation because corneal epithelium represents one of the major infection sites for HSV-1 and may precede infection of other parts within the eye [28]. Plaquing efficiency of HSV-1 was examined in HCE cells after either overexpressing or downregulating syndecan-1. Confluent monolayers of HCE overexpressing syndecan-1, or have syndecan-1 downregulated were infected with serial dilutions of virus stocks and overlaid with 0.5% methylcellulose growth medium. Several days post-infection, the monolayers were fixed and stained and plaques were counted. Surprisingly, the number of plaques formed after syndecan-1 overexpression on HCE cells resulted in an insignificant reduction in plaque formation. However, as seen with cell fusion assay, the downregulation of syndecan-1 resulted in 57.37±3.96 % reduction in plaques formation (*p*<0.0001) (Fig. 5B). Overexpressing syndecan-1 mutants did not show significant difference in plaque number. However one mutant showed smaller plaque size.

### Increase in syndecan-1 production increases plaque size, while a reduction in its expression and overexpression of a syndecan-1 mutant that lacks the ectodomain form smaller size plaques

**Figure 6:**
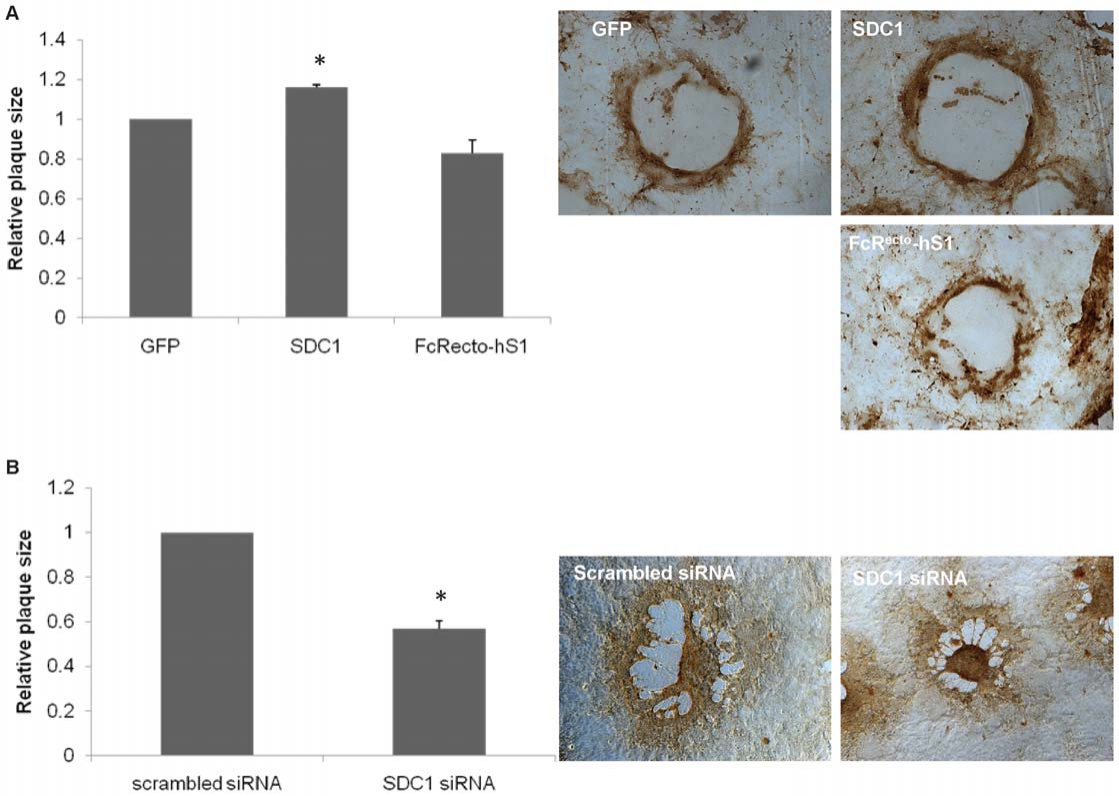
Effect of syndecan-1 overexpression, or downreguulation on HSV-1 plaque size. Monolayers of HCE cells were transfected with either control GFP plasmid, full-length *wt* syndecan-1 (SDC1), or the construct FcR^ecto^hS1. 24 h post-transfection, cells were infected with HSV-1 (KOS) at an MOI of 0.1 and overlaid with 0.5% methylcellulose medium. (B) 50% confluent HCE cells were transfected with either control scrambled siRNA or syndecan-1 sprecific siRNA. 72 h post-transfection, cells were infected with HSV-1 (KOS) at an MOI of 0.1 and overlaid with 0.5% methylcellulose medium. (A, B). 72 h post-infection cells were fixed and stained using rabbit anti-HSV-1, horseradish peroxidase-conjugated secondary antibody, and 3,3′-diaminobenzidine tetrahydrochloride (DAB) substrate. Plaques were measured with a micrometer at the 10× objective (Zeiss Axiovert 200), and the area was calculated by measuring the outline of each plaque using Axioversion software (version 4). Results are expressed as relative plaque size (means ± 1 SD) of two independent experiments conducted in duplicate. Representative plaques from each condition are shown. *SDC1*, syndecan-1.

To further investigate the role of syndecan-1 in lateral HSV-1 transmission, plaque size was examined after overexpressing wild type syndecan-1 and syndecan-1 mutants, or downregulating syndecan-1 in HCE cells. As demonstrated in Fig. 6A, the average plaque size formed in HCE cells after syndecan-1 overexpression was larger than that of the positive control HCE cells that express normal levels of syndecan-1 (16.19%±1.13, *P<*0.05), showing that wild type syndecan-1 induced lateral virus spread, confirming the cell fusion results where enhancement of syndecan-1 production showed increase in cell fusion which is one major way the virus can laterally spread (Fig. 6A). Interestingly, overexpressing the mutant FcR^ecto^-hS1 that lacks the ectodomain of syndecan-1 showed smaller size plaques compared to the positive control (17.16%±6.54, *P* = 0.065) indicating that the ectodomain of syndecan-1 might play a role in the virus lateral spread (Fig. 6A). Representative plaques from each condition are shown in Fig. 6A. Since the increase insyndecan-1 resulted in larger size plaques, the effect of syndecan-1 loss on plaque size was examined. Syndecan-1 downregulation reduced plaques sizes 42.92%±3.27 (*P*<0.05) compared with HCE cells transfected with the control scrambled siRNA supporting the involvement of syndecan-1 in HSV-1 lateral cell-to-cell spread (Fig 6B). Representative plaques from each condition are shown in Fig 6B.

### Syndecan-1 downregulation reduces HSV-1 spread

**Figure 7:**
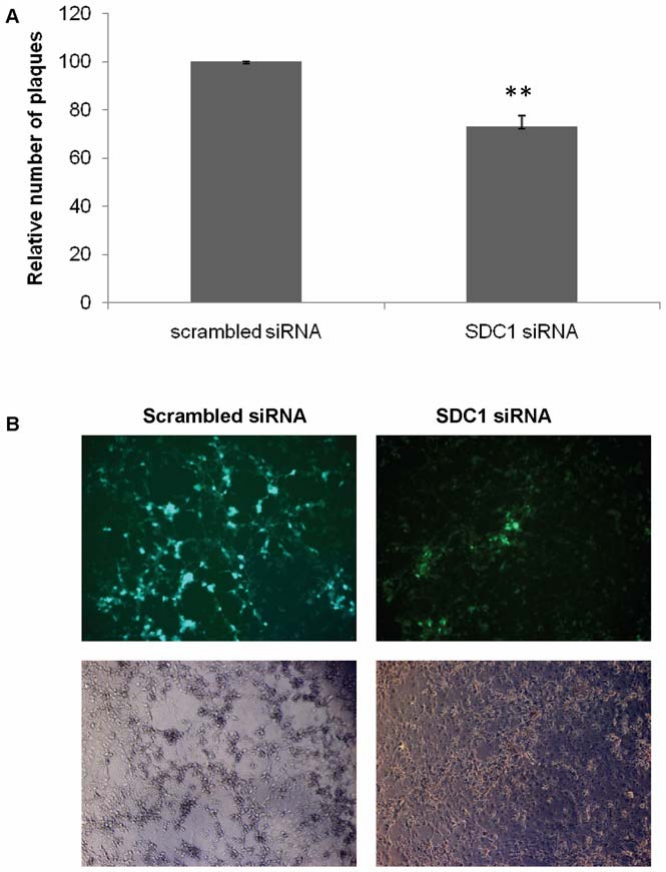
Syndecan-1 downregulation reduces HSV-1 spread. Monolayers of HCE cells were exposed to (A) HSV-1 (KOS) or (B) HSV-1 (KOS) K26GFP strains at MOI of 5 at 37°C to allow virus entry. 2 h post-infection, cells were washed, incubated for 1 min with 0.1 M citrate buffer (PH 3.0), then washed with PBS and overlaid with 0.5% methylcellulose medium. After 4 h, cells were washed, dissociated, and approximately 600 cells were plated onto 80% confluent monolayers of uninfected HCE cells that have been transfected with either scrambled siRNA or syndecan-1 siRNA for 72 h in 0.5% methylcellulose medium. (A). The spread of HSV-1 (KOS) from infected cells to the siRNA transfected HCE cells was evaluated by staining and counting the number of plaques formed at the 10× objective (Zeiss Axiovert 200). Results are presented as relative number of plaques (mean ± 1SD) of four independent experiments performed in duplicates. (B) HSV-1 (KOS) K26GFP spread was qualitatively assessed at the 10× objective (Zeiss Axiovert 200). Representative images from one experiment performed in triplicate are shown. *SDC1*, syndecan-1.

Since syndecan-1 downregulation showed significantly smaller plaque size compared to control cells transfected with scrambled siRNA, we aimed to confirm the involvement of syndecan-1 in virus lateral cell-to-cell spread by performing virus spread assay. HSV-1(KOS) infected HCE cells, which were treated with low pH citrate buffer to inactivate residual virus, were mixed with syndecan-1 siRNA transfected cells, and the virus spread was evaluated by counting the number of plaques formed 48–72 h post mixing. Syndecan-1 knockdown resulted in less virus spread compared to HCE cells transfected with scrambled siRNA (26.93±4.62, *P*<0.0001) (Fig. 7A). A qualitative fluorescent approach was also utilized to examine the effect of syndecan-1 downregulation on virus spread. HSV-1 (KOS) K26GFP virus strain that has the jellyfish green fluorescent protein (GFP) fused in frame with the UL35 open reading frame generating K26GFP virus whose capsids express GFP [35] was used in the spread assay (Fig.7B). The top panels show representative HSV-1 (KOS) K26GFP virus spread to HCE cells treated with either syndecan-1 siRNA or the control scrambled siRNA. Syndecan-1 downregulation resulted in less virus spread as indicated by the less green fluorescence compared to control scrambled siRNA transfected cells. The bottom panels show HCE cells under the bright field. Together these results strengthen the conclusion that syndecan-1 is involved in HSV-1 lateral cell-to-cell spread.

### Syndecan-1 downregulation reduces infectious virus production

**Figure 8:**
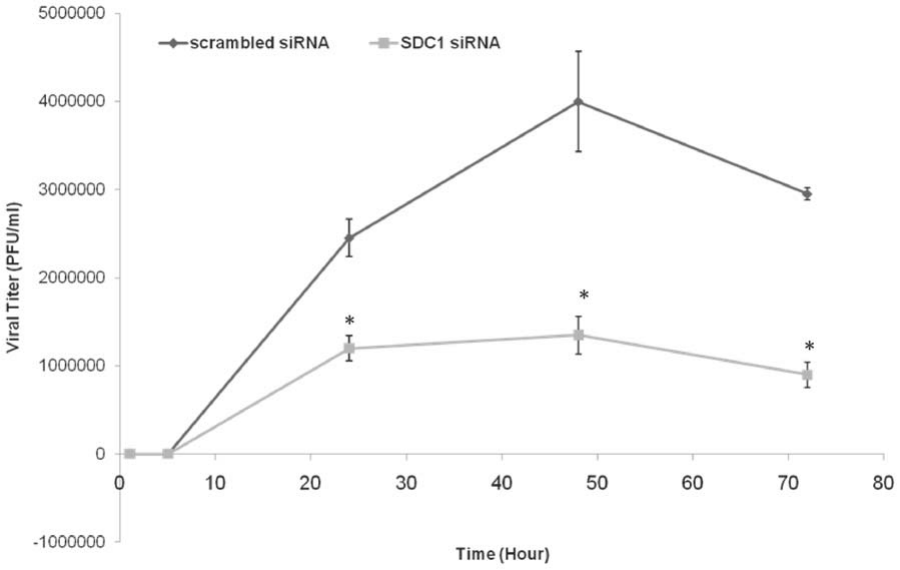
Downregulation of Syndecan-1 results in reduced production of infectious virus. HCE cells were transfected with either control scrambled siRNA or syndecan-1 siRNA. 72 h post-transfection cells were infected with HSV-1 (KOS) at an MOI of 0.1. At 0, 5, 24, 48, and 72 h post-infection, infectious virus was quantified by a standard plaque assay on HCE cell monolayers. The titers shown are the mean ± 1 SD of a representative experiment of two independent experiments performed in duplicates. *SDC1*, syndecan-1.

Since knocking down syndecan-1 on HCE cells resulted in reducing the plaquing efficiency of HSV-1, we sought to verify whether this reduction also translates into a loss of infectious virus produced as well. To answer this question, infectious virus production was analyzed after syndecan-1 downregulation. HCE cells transfected with either scrambled siRNA or syndecan-1 specific siRNA were infected 72 h post-transfection with HSV-1 (KOS) at an MOI of 0.1. At various times post-infection, cells and media were harvested, and virus titers were measured. Consistent with the results of plaquing efficiency, the downregulation of syndecan-1 severely impaired infectious virus production (*P<*0.05) (Fig. 8). Taken together, these results suggest that downregulation of syndecan-1 also negatively impacts infectious virus production.

## Discussion

HSV-1 entry into host cell starts with the attachment of HSV-1 gB and gC with HSPG on the surface of the host cell. While substantial work has focused on delineating the role of HS as an attachment receptor for HSV-1, far less is known about the contribution of the HSPG core protein in HSV-1 infection. Our study demonstrates a novel role for the core protein of HSPG during HSV-1 infection. Specifically, we demonstrate that the core protein of syndecan-1 contributes to HSV-1 induced cell-to-cell fusion and lateral spread and those functions are independent of HS chains. The increase of syndecan-1 in target cells enhanced HSV-1 induced cell-to-cell fusion, while its decrease reduced cell fusion as well as virus spread.

A related interesting finding of our study is that the overexpression of syndecan-1 in effector cells that express HSV-1 fusion glycoproteins (gD, gB, gH, and gL) showed significantly reduced HSV-1 induced cell-to-cell fusion. The observed reduction in cell-to-cell fusion is reminiscent of a previously described phenomenon called gD-mediated interference, where expressing gD in HSV-1 susceptible cells results in resistance to viral entry due to sequestering gD receptors and preventing their accessibility by cell-associated gD [19], [36]. This raises the possibility that there might be a direct interaction between syndecan-1 and at least one of the fusion glycoproteins, which in turn sequester at least one of the glycoproteins preventing them from being fully functional during cell-to-cell fusion. Our results showed similar levels of inhibition of cell-to-cell fusion in both CHO-K1 and CHO-745 cells. The latter differ from CHO-K1 cells in that CHO-745 cells are deficient in the synthesis of all GAGs [30], [31]. This suggests that this inhibition in cell-to-cell fusion is HS independent and not due to interactions between HS and HSV-1 fusion glycoproteins, since it is well known that gB utilizes HS chains on HSPGs as an attachment receptor to initiate the entry process of the virus into the host cell [8].

Another possibility is that syndecan-1 affects indirectly by physically binding other cellular protein(s) that HSV-1 glycoprotein(s) require for fusion. This binding could also prevent the cellular protein from interacting with HSV-1 fusion glycoproteins, and that somehow leads to less efficient cell-to-cell fusion. Syndecan-1 ectodomain and cytoplasmic domain, which our results indicate their importance in this phenomenon, are both been shown to interact with a wide range of cellular proteins [37]. For example syndecan-1 ectodomain has been shown to interact directly with β3 or β5 integrins, which in turn have been shown to be cellular determinants of HSV-1 rout of entry [38], [39]. It is also possible that overexpressing syndecan-1 along with HSV-1 fusion glycoproteins may perturb cellular signaling mechanisms required for efficient cell-to-cell fusion. The core protein of syndecan-1 functions in a variety of signaling pathways that are involved in regulatory events, like actin cytoskeleton reorganization, cell adhesion, cell proliferation, and angiogenesis, many of which are involved in HSV-1 infection [26], [40]–[48]. Further work will be critical to clearly understand the reason behind the reduced cell-to-cell fusion upon syndecan-1 overexpressing along with HSV-1 fusion glycoproteins in effector cells.

This study also demonstrates that syndecan-1 is involved in HSV-1′s ability to form plaques. Although syndecan-1 overexpression did not result in increased number of plaques, the knockdown of syndecan-1 resulted in significant impairment in plaques formation. One possible explanation is that syndecan-1 might be a regulatory component of a multi-protein complex that affects virus plaque formation. In that case, loss of syndecan-1, and not so much the extra copies of syndecan-1, is critical for cell-to-cell fusion. It is also possible that for this multi-protein complex, over-expression of syndecan-1 alone may not lead to more complex formation since the other components of that complex are not overexpressed. Another explanation is that syndecan-1 is involved in signaling pathways important for virus plaque formation, so a reduction in syndecan-1 level resulted in less efficient plaque formation, but an enhancement in syndecan-1 production did not affect the virus's ability to form plaques.

Although the overexpression of syndecan-1 did not result in more efficient plaque forming ability of HSV-1, it did, however, result in plaques with bigger sizes. By the same token, the knockdown of syndecan-1 resulted in smaller size plaques compared to control siRNA transfected cells. The plaque assays were preformed in the presence of methylcellulose layer where plaque formation depended exclusively on cell-to-cell virus spread. This suggests that syndecan-1 is playing a key role during HSV-1 virus spread. This suggestion was strengthen by a spread assay where the knockdown resulted in less virus spread as evident by a quantitative and a qualitative spread assays.

We further analyzed the effect of syndecan-1 knockdown on infectious virus titers after HSV-1 (KOS) infection utilizing HSV-1 growth curve assay. Syndecan-1 knockdown severely reduced the titers of infectious virus. The effect of syndecan-1 downregulation on HSV-1 growth curve could be an accumulative effect of syndecan-1 playing role in HSV-1 induced cell fusion, HSV-1 spread and its role in virus entry suggested by our lab and others [10], [11]. However, this does not exclude the possibility that at least part of this observed reduction in HSV-1 growth curve might be due to less HSV-1 replication in HCE cells transfected with syndecan-1 siRNA.

Further studies must be done to determine the molecular mechanisms behind the contribution of syndecan-1 during HSV-1 infection. The emerging role of syndecan-1 during HSV-1 infection opens new doors that might add clarity to the picture of virus entry and infection. The role of syndecan-1 has been investigated during other virus infections including Kaposi's sarcoma-associated herpesvirus [49], [50], human papillomaviruses [51], and human immunodeficiency virus (HIV) infection, where, in the case of HIV infection, syndecan-1 has been shown to be involved in capturing and transmitting the virus to permissive cells [52]. Delineating the role of syndecan-1 during the various HSV-1 infection events, especially those at the early stages of the infection is of significance as that might help the development of new antiviral agents or an effective HSV-1 vaccine.

## Materials and Methods

### Cell culture and viruses

Wild type Chinese hamster ovarian (CHO-K1) cells, mutant CHO-745 cells, and Vero cells were provided by P. G. Spear (Northwestern University). The human corneal epithelial (HCE) cell line (RCB1834 HCE-T) was provided by Dr. Kozaburo Hayashi (National Eye Institute, Bethesda, MD) [53]. All CHO cell lines were grown in Ham's F-12 medium (Gibco/BRL, Carlsbad, CA, USA) supplemented with 10% fetal bovine serum (FBS) and penicillin and streptomycin (P/S) (Sigma). Vero cells were grown in Dulbecco's modified Eagle's medium (DMEM) supplemented with 10% FBS and P/S. HCE cells were grown in minimum essential medium (MEM) supplemented with 10% FBS and P/S. Wild type HSV-1 (KOS) virus strain was provided by P.G. Spear (Northwestern University). HSV-1 (KOS) K26GFP virus strain was provided by P. Desai (the Johns Hopkins Universiy) Jellyfish green fluorescent protein (GFP) was fused in frame with the UL35 open reading frame generating K26GFP virus whose capsids express GFP [35]. Virus stocks were propagated and titered on Vero cells, and stored at−80°C.

### Plasmids and Antibodies

HSV-1 (KOS) glycoproteins expressing plasmids used were pPEP98 (gB), pPEP99 (gD), pPEP100 (gH), and pPEP101 (gL) [54]. Wild type human syndecan-1 (SDC1) and human syndecan-1 mutants including FcR^ecto^hS1 (a chimera comprised of the ectodomain of human IgG Fcγ receptor Ia/CD64 fused to the transmembrane and cytoplasmic domains of human SDC1), hS1^Δcyto^ (lacking the 33 C-terminal amino acids) and hS1^pLeu^™ (transmembrane domain replaced with leucine residues) were provided by Alan Rapraeger (University of Wisconsin-Madison) [55]. The following antibodies were used for this study: Rabbit anti-syndecan-1 polyclonal antibody (Ab) diluted 1∶500 (sc-5632 Santa Cruz Biotechnology, Santa Cruz, CA); mouse anti-β-actin monoclonal Ab (mAb) at 1∶1000 dilution (A-5316 Sigma-Aldrich); mouse anti-syndecan-1 mAbs at 1 mg per 1×10^6^ cells dilution (Santa Cruz Biotechnology). Secondary Abs for Western blots were horseradish peroxidase-conjugated goat anti-rabbit IgG diluted 1∶20000 (73102, Jackson ImmunoResearch Laboratories, West Grove, PA); and horseradish peroxidase-conjugated anti-mouse IgG diluted 1∶25000 (115-035-062, Jackson ImmunoResearch Laboratories). Secondary Ab for flow cytometry was FITC-conjugated anti-mouse secondary antibody diluted 1∶100 (Sigma-Aldrich).

### siRNA and DNA plasmid Transfections

For human syndecan-1 (accession number NM_002997) knock-down experiments, two siRNAs generated with 3′-dTdT overhangs and prepared by Sigma, were chosen against the DNA target sequences as follows: (5′-CCATTCTGACTCGGTTTCT-3′, 5′-GCCAAGGTTTTATAAGGCT-3′). siRNA cell transfection was performed using Lipofectamin 2000 reagent (Invitrogen, Cergy Pontoise, France) with 200 nM duplex siRNA according to the manufacturer's recommendations. In all experiments, cells were analyzed 72 h after transfection. A nonspecific scrambled siRNA (GAUCAUACGUGCGAUCAGA) was used as a negative control (Sigma). For DNA plasmid transfection, cells were grown to 80% confluency, and Lipofectamin 2000 reagent was used according to the manufacturer's recommendations.

### Immunoblotting

72 h post-transfection with syndecan-1 siRNA, or scrambled siRNA, syndecan-1 protein expression was determined by Western blot analysis. The Western blot assay was performed according to protocols described previously [56]. Briefly, Whole cell lysates of the siRNA-transfected cells were denatured in NuPAGE LDS Sample Buffer (Invitrogen, NP0007) and heated to 86°C for 8 min before gel loading. Equal amounts of protein were subjected to 4–12% SDS-PAGE and electroblotted onto a nitrocellulose membrane. Nonspecific binding was blocked using 5% nonfat milk in tris buffered saline (TBS) for 2 hours at 37°C. The membranes were then incubated with primary rabbit polyclonal antibodies (Santa Cruz) to sydecan-1 at 1∶500 dilutions overnight at 4°C. The blots were rinsed 5 times with 0.1% TTBS (0.1% Tween 20 in TBS) for 5 min followed by incubation for 1 h at room temperature with horseradish peroxidase-conjugated anti-rabbit IgG (Promega, 1∶20000). Protein bands were detected using SuperSignal West Femto maximum sensitivity substrate (Pierce, 34096), and visualized using ImageQuant LAS 4000 imager (GE Healthcare Life Sciences). Protein bands were quantified using ImageQuant TL image analysis software (version: 7). For repeated probing, the blots were stripped for 30 min at room temperature with Restore™ Western Blot stripping buffer (Thermo scienctific, 21059). β-actin was measured as a loading control. Syndecan-1 was quantified by calculating the relative intensity of each syndecan-1 band to that of β-actin.

### Flow cytometry

Syndecan-1 cell surface expression was detected after syndecan-1 transfection in CHO-K1, CHO-745, and HCE cells. Confluent monolayer of cells were either mock treated or transfected with human syndecan-1 plasmid for 48 h. Cells were then washed with PBS, harvested, and incubated with syndecan-1 primary antibody at 1 mg per 1×10^6^ cells diluted in PBS with 1% BSA for 1 h at 4°C. After primary antibody incubation, cells were washed and incubated for 30 min with anti-mouse-FITC-conjugated secondary anti-IgGs (1∶100). Cells stained only with anti-mouse-FITC were used as background control.

### Cytotoxicity assays

For cytotoxicity studies after syndecan-1 and syndecan-1 mutants overexpression, 2.5×10^4^ cells/well were plated in 96-well plates and tranfected with full-length *wt* syndecan-1, the construct FcR^ecto^hS1, the construct hS1^Δcyto^, the construct hS1^pLeu^™ or control GFP plasmid. For cytotoxicity studies after syndecan-1 knockdown, 1×10^4^ cells/well were plated in 96-well plates and tranfected with syndecan-1 siRNA, or scrambled siRNA. After 6 h of transfection, serum-enriched medium was added and cultures were followed for 24 h after overexpressing syndecan-1 and 72 h after downregulating syndecan-1. Cell viability was evaluated by a chromogenic kit (CellTiter AQueous96; Promega, Madison, WI, USA) and colorimetric detections were performed using microplate ELISA reader (Spectra Max 190 Molecular Devices, Sunnydale, CA USA). Results were expressed as 100% wild type (wt) viability where they represent the percent corrected absorbance after subtracting the background absorbance, relative to GFP transfected cells in syndecan-1 overexpression experiments, or relative to scrambled siRNA transfected cells in syndecan-1 downregulation experiments.

### Cell-to-Cell Fusion Assays

Standard cell-to-cell fusion assay was used as previously described [30], [57]. Cells were split into two populations. “Target” cells were transfected with plasmid expressing Nectin-1 as a gD receptor (1.0 µg) and the luciferase gene (0.5 µg). “Effector” cells were transfected with plasmids expressing HSV-1 glycoproteins gD, gB, gH, and gL and T7 RNA polymerase (0.5 µg each). For cell fusion experiment after syndecan-1 overexpression, target or effector cells were additionally transfected with 0.5 µg of a plasmid expressing human syndecan-1 or control green fluorescent protein (GFP) plasmid. For cell fusion experiment after syndecan-1 knockdown, target cells were first either mock treated or transfected with syndecan-1 siRNA. After 24 h, target and effector cells were transfected as described above. After 16 h, target and effector cells were mixed in a 1∶1 ratio and replated in 24-well dishes. Luciferase activity was measured after 16 h. As a negative control, target cells were mixed with effector cells that lack HSV-1 gB.

### Syncytia Assay

Assay was performed as previously described [26]. Target cells were additionally transfected with 0.5 µg of a plasmid expressing cyan fluorescent protein (CFP) fused to a nuclear localization signal (NLS) (Clontech, Mountain View, CA). Effector cells were also transfected with a red fluorescent protein (RFP)-expressing plasmid fused to a nuclear export signal (NES) [32]. Target and effector cells were mixed in a 1∶1 ratio and replated in 8-chamber slides (Lab-Tek Corp.). Syncytia images were captured after 72 h using microscopy at the 40× objective on a confocal microscope (Leica DMIRE2) equipped with a camera (Leica TCSSP2). Syncytia size and number were compared after 72 h at the 10× objective (Zeiss Axiovert 200). Number of syncytia was normalized to the number of syncytia detected in the negative control wells where the effector cell population lacks gB.

### Virus growth assays

#### Plaque assays

Monolayer of HCE cells in 24 well plates overexpressing syndecan-1, syndecan-1 mutants, or GFP control plasmid (0.8 µg using Lipofectamine 2000 reagent), or transfected with syndecan-1 siRNA, or scrambled siRNA, as described above, were infected with 10-fold serial dilutions of HSV-1 (KOS) virus stocks. Infected cells were fixed with methanol, stained with crystal violet, and plaques were counted at the 10× objective (Zeiss Axiovert 200).

#### Plaque size determination

Monolayer of HCE cells in 4-well chamber slides (Lab-Tek Corp.) overexpressing syndecan-1, syndecan-1 mutants, or GFP control plasmid, or monolayer of HCE cells in 24-well plate transfected with syndecan-1 siRNA, or scrambled siRNA, were infected with 0.1 MOI of HSV-1 (KOS). After 2 h of adsorption at 37°C, inoculums were removed; cells were washed twice with PBS. Infected cells were overlaid with 0.5% methylcellulose (sigma). The overlay was removed at 3 dpi, and cells were fixed with a 1∶1 dilution of methanol:acetone, or 100% methanol. Plaques were stained using rabbit anti-HSV-1 (Dako), horseradish peroxidase-conjugated secondary antibody (Amersham), and 3, 3′-diaminobenzidine tetrahydrochloride (DAB) substrate (Sigma). Plaques were measured with a micrometer at the 10× objective (Zeiss Axiovert 200), and the area was calculated by measuring the outline of each plaque using Axioversion software (version 4). The average area was determined by measuring 22 plaques from each of the two experiments performed in duplicate.

#### Growth curves

Monolayers of HCE cells in 6 well plate transfected with syndecan-1 siRNA, or scrambled siRNA, were infected with HSV-1 (KOS) at a multiplicity of infection (MOI) of 0.1. After 2 h of adsorption at 37°C, inoculums were removed, cells were washed twice with Hanks' balanced salt solution, and growth medium was added back to each culture. 1, 5, 24, 48, and 72 h post-infection, cells were scraped into medium, and cell suspensions were transferred to tubes, sonicated, clarified by centrifugation (800× *g* for 10 min), and stored at −80°C. Infectious virus in the supernatant was quantified by standard plaque assay on HCE cell monolayers.

### Virus spread Assay

Virus spread assay was designed using previously described protocols [58], [59]. Monolayer of HCE cells were exposed to HSV-1 (KOS) or HSV-1 (KOS) K26GFP strains at MOI of 5, at 37°C to allow virus entry. 2 h post-infection, cells were washed once with PBS, and then incubated for 1 min with 0.1 M citrate buffer (PH 3.0) to inactivate residual virus particles. Cells were then washed three times with PBS and overlaid with 0.5% methylcellulose (sigma) MEM medium. After 4 h, cells were washed three times with PBS, and dissociated using Hanks'-based enzyme free dissociation buffer (Invitrogen). Approximately 600 cells were plated onto 80% confluent monolayers of uninfected HCE cells that have been transfected with either scrambled siRNA or syndecan-1 siRNA for 72 h in 0.5% methylcellulose MEM medium. The spread of HSV-1(KOS) from infected cells to the siRNA transfected HCE cells was evaluated by staining and counting the number of plaques formed at the 10× objectives (Zeiss Axiovert 200). Whereas the spread of HSV-1 (KOS) K26GFP from the infected cells to the siRNA transfected HCE cells was qualitatively assessed by capturing images of the green virus at the 10× objective (Zeiss Axiovert 200).

### Statistical analyses

The data shown are the means ± 1SD values. Statistical analyses were performed with GraphPad Prism software (version 4.0). Data were assessed using unpaired student's *t* test. *P<0.05 and **P<0.0001 were regarded as significant differences between treated and mock-treated groups.
